# Making ‘*being less sedentary feel normal’* –investigating ways to reduce adolescent sedentary behaviour at school: a qualitative study

**DOI:** 10.1186/s12966-023-01444-y

**Published:** 2023-07-11

**Authors:** Anne-Maree Parrish, Anthony D. Okely, Jo Salmon, Stewart Trost, Megan Hammersley, Anisse Murdoch

**Affiliations:** 1 School of Health and Society, Faculty of Arts Social Science and Humanities, University of Wollongong, Wollongong, Australia; 2 Early Start, University of Wollongong, Wollongong, Australia; 3 School of Exercise and Nutrition Sciences, Faculty of Health, Deakin University, Melbourne, Australia; 4 School of Human Movement and Nutrition Sciences, Faculty of Health and Behavioural Sciences, University of Queensland, Brisbane, Australia

**Keywords:** Adolescents, Secondary schools, Sitting, Sedentary time

## Abstract

**Background:**

Adolescents spend over 50% of a 24-hour period and 63% of the school day sedentary. Few comprehensive qualitative studies have explored teachers’ and students’ perceptions of potential strategies to reduce sedentary behaviour (SB) in the secondary school setting. This project aimed to elicit students’ and teachers’ perspectives of feasible and acceptable ways to encourage adolescents to “sit less and stand or move more” during the school day.

**Methods:**

Students, teachers, and executives from four schools in the Illawarra and surrounding areas (New South Wales) Australia, were invited to participate. Focus group implementation used a participatory research design (‘problem and solution tree’). Participants were interviewed in three groups, younger adolescents, older adolescents and teachers/executives. Firstly the ‘problem’ (high rates of SB) was explained, participants were then asked to identify contributing school related factors, and to suggest feasible ideas to reduce SB during the school day.

**Results:**

Fifty-five students (24 from Years 7/8 aged 12–14 years and 31 from Years 9/10 aged 14–16 years), and 31 teachers consented to participate. Thematic analysis elicited five main ‘problems’: lesson structure, non-conducive classroom environment/structure, non-conducive break-time environment, curricular pressures and school-related factors increasing sedentary behaviour outside of school. Suggested ‘solutions’ included: changes to classroom layout/furniture, pedagogical changes, hands-on learning, outdoor lessons, more comfortable uniforms, more breaks during class time, compulsory physical activity, and outdoor equipment.

**Conclusions:**

The proposed solutions to reduce adolescent SB during the school day have potential to be feasibly implemented in the school setting, even with limited funding.

**Supplementary Information:**

The online version contains supplementary material available at 10.1186/s12966-023-01444-y.

## Background

The rapid evolution of electronic screen based activities (television, smart phones, iPads’ and computers) has influenced children’s and adolescents’ discretionary time, increasing levels of sedentary behaviour (SB) [1]. SB is defined as ‘having a Metabolic Equivalent of Task (MET) value between one and 1.5 (for example, equivalent to sitting or lying down)’ [2]. High levels of SB are associated with cardiometabolic risk, unfavourable body composition, poor conduct/prosocial behaviour, reduced fitness, lower self-esteem, anxiety and depression in children and adolescents [3].

There is growing evidence supporting the benefits of substituting SB with both light (LPA) and moderate- to vigorous-intensity physical activity (MVPA) [4]. Moura and colleagues [4] investigated the cardiometabolic effects of isotemporal substitution of SB with LPA in male adolescents, reporting a positive effect on high-density lipoprotein cholesterol (HDL-C), insulin sensitivity - Homeostasis Model Assessment 2 (HOMA2-S) and systolic blood pressure (SBP). Reducing SB may also positively affect adolescents’ cognitive function [5].

SB substantially increases from childhood to adolescence. Adolescents spend over 50% of their total day and on average 63% of the school day sedentary [6]. A pooled analysis of international children’s accelerometry data found children’s and adolescents’ sedentary time increased by 21.4 min/day for each year as they age [7]. The transition from primary/elementary to secondary school increases children’s SB by 58 min per day [8]. Given this dramatic increase in sedentary behaviour, secondary school may be the optimal life stage to reduce the excessive and increasing amount of SB during adolescence.

A systematic review and meta-analysis of school based interventions to promote physical activity (PA) and/or reduce SB in children found no evidence of an effect on MVPA and inconclusive evidence of an effect on SB [9]. The interventions included ‘class PA breaks, physically active learning, before- and after-school clubs, physically active homework, active travel, and a whole-school PA policy’ but did not focus specifically on reducing SB.

Whilst efforts to promote MVPA are important, there could be more opportunities during the school day to incorporate increases in LPA in the classroom setting. Implementing school policy changes to reduce SB has generally been successful in primary/elementary schools [10]. A systematic review of interventions examining the impact of school-based standing desks on children’s SB in primary/elementary schools found integrating standing desks reduced SB by approximately 30 min per day [11]. Other studies have reduced SB through regular activity breaks [9] or through the use of flexible learning spaces [12]. However, barriers associated with implementing school-based SB (under resourcing, under qualified staff, time constraints, school curricular priorities) can hinder research translation [13]. SB interventions which incorporate measures of academic performance (such as executive function, time on task, class management) could make SB interventions a higher priority for teachers, principals and education authorities, thereby increasing the feasibility, acceptability and adoption of these interventions [14].

Research in the secondary (middle) school setting is limited. One study demonstrated significant reductions in adolescents’ school day SB using height-adjustable desks [15]. Another surveyed teachers to understand their perceptions of using standing desks and activity breaks in primary/secondary school and university settings. [16]. Whilst there were concerns the desks could increase class disruption, most teachers believed standing desks and physical activity breaks were feasible in the classroom setting. A study in college and vocational education settings explored the use of standing desks [17] finding that students needed to be motivated by teachers to use the desks, even when they were aware of the health benefits of reducing SB [17]. It is critical to identify potential strategies to reduce SB during adolescence in the secondary setting due to the paucity of research in this area.

This study is informed by both a systematic and systemic approach; acknowledging the complexity of SB and the contribution of both schools of thinking [18]. Systematic approaches assist in categorising and conceptualising determinants associated with sedentary behaviour, situating the individual at the centre of muliple layers of influence [19]. The socioecological model posits that adolescents are influenced by their interpersonal relationships with peers and teachers (microsystem), the school community including the school ethos and pedagogies (mesosystem), the organisational level (exosystem) which considers the physical environmental, classroom including layout, furniture and resources and playground design, and the outer layer is the policy level that includes the influence of school policies and curriculum (macrosystem) [19]. Whereas systems thinking is a social learning process, requiring ongoing enquiry to transition from the problematic behaviour (SB) to the a heathier state. Systems thinking considers the subsystems of policy-making and sectors which may influence SB [20]. Systemic frameworks such as the SOS-framework (System of Sedentary behaviours) extends beyond the limitations of the socioecological model removing the focus on the individual and considers the health and wellbeing of “individuals and groups, their psychology and behaviour, culture and social context, the built and natural environment, the institutions and the politics and economics” [18].

To date, few studies have explored teachers’ and students’ suggestions to reduce SB in the secondary school setting. Focus groups are a commonly used method to understand the perceptions of both adults and adolescents [21]. However adolescents’ developmental needs can add to the complexity of this method [21]. Challenges include their “need for peer approval, declining social trust, short attention span, and reliance on concrete operations thinking” [21]. A ‘problem and solution tree’ approach is a simple qualitative participatory tool which increases stakeholder awareness, is easy to implement, involves participants in the identification of issues and potential solution which can lead to a wealth of information [22]. This method could alleviate some of the complexities of adolescent focus groups.

This study aimed to investigate teachers’ and students’ suggestions of feasible and acceptable ways to encourage adolescents to “sit less and stand or move more” during the school day in order to improve adoption and implementation of future SB interventions in the secondary school setting using a novel methodology.

## Methods

### Recruitment

A publicly available online list of 464 independent secondary schools in New South Wales, Australia was obtained [23]. “Independent schools are non-government schools and they include catholic schools, they operate autonomously, are registered, and their teachers accredited by the NSW Education Standards Authority, they are educationally and financially accountable to their Boards, and the Australian and NSW Governments” [23]. The 38 schools that were contacted as part of this research were a convenience sample based on geographical location nearest to the University. The schools were contacted via phone and email between 2013 and 2014 until four schools were recruited (the number of participating schools was limited by funding contraints). Three schools included students from Kindergarten to year 12, one school was a single sex secondary school. The size of the secondary school cohorts ranged from approximately 50 to 880 students (see Supplementary material 1, supplementary table 1).

### Ethics and consent

The school principal from consenting schools was sent information sheets and consent forms via email which were then distributed to students via the schools normal process for note distribution. Information sheets were distributed to teachers and executive staff during a staff meeting or via their internal mail boxes. Consent was received prior to the commencement of the study at each school. All schools were offered the opportunity for the research staff to explain the study to staff and students in person prior to implementation (one school accepted this offer). Children who were older than 16 years of age provided their own consent to participate in the study, children under 16 years of age acquired parental consent and gave assent prior to participating in the focus groups. Verbal consent was obtained from staff who chose to be involved in the focus groups. The focus groups took place at a time that suited each school schedule, and they were conducted in a space designated by each school (e.g., small room near the staff room). The study was approved by the University of Wollongong Human Research Ethics Committee (HE13/277).

### Instrumentation

This research explored activities and postures constituting a current “typical” school day (i.e., around 50% SB) and an ideal “sit less and stand or move more” school day (around 25% time sedentary i.e., a 50% reduction in SB). The study used a participatory research design known as the ‘problem and solution tree’, to guide the focus group implementation [22]. Snowdon defines a problem and solution approach as a “participatory process of working through the layers of determinants and then developing potential interventions for a specific issue, using the available data and expertise” [22]. This methodology firstly involves asking participants to recognise why a problem occurs, what factors contribute to the problem, and what the potential consequences of the problem are. The process then involves participants considering potential solutions (or objectives) to the ‘problem’. In relation to the current study, we asked participants to change the problem of ‘high levels of SB’ during the school day to a solution orientation by providing options to reduce SB. Participants were then asked to consider the potential effects of the proposed solutions [22].

### Development of the focus group script

The research team developed a focus group script based on the problem solution tree [22]. The research team practiced the script then trialed it in the pilot study. Overall, it was found to be acceptable during the pilot study, although slight changes were made to make is more concise. A secondary school teacher verified its appropriateness for the adolescent cohort prior to the main study, no changes were made to the version reviewed (see Supplementary Material 2).

### Pilot study

This study was preceeded by a pilot study with a convenience sample of two focus groups of secondary school students (one with younger -Years 7, 8 and 9 and one with older adolescents Years 10, 11 and 12) in regional New South Wales, Australia. These age groupings were chosen to ensure younger secondary students did not feel intimidated by their older counterparts and to give them a voice. Following the pilot and after discussion with school staff involved in the study, it was decided recruitment should exclude Year 11 and 12 students who were involved in exams during the data collection period.

The pilot was designed to assess the feasibility of the ‘problem/solution tree’ methodology prior to commencing the study [22] (including the questions and script) and to train the research assistants. Four researchers were trained to conduct the focus groups. They practiced the script and method prior to implementing the pilot, where they took turns assisting in the two roles (leading and recording the discussion).

### Data collection

Data collection commenced in October 2013 and concluded in April 2014. Three focus groups were conducted at each of the four schools at prearranged times to accomodate school schedules (A total of 12 focus groups). One included younger students (Years 7, 8 – aged between 12 and 14), a second included older students (Years 9 and10 – aged between 14 and 16) and a third included teachers and executive staff. Each focus group involved between three and ten participants (only one focus group had less than 5 participants). The focus groups took approximately 30 to 45 min to conduct.

### Focus groups

At the commencement of each focus group students and teachers were presented with current evidence regarding adolescent SB during the school day and the health consequences of prolonged and uninterrupted SB, consistent with government guidelines. Using the ‘Problem-and Solution tree tool’, each focus group followed these steps: (1) A discussion about the high rates of prolonged SB for adolescents; (2) The identification of factors (e.g.: school policy, physical and social environment) participants believe contribute to this problem in the school environment and related health and educational consequences; (3) Brainstorming positive solutions to the causes of the identified factors; (4) Prioritisation of identified solutions based on feasibility and acceptability; (5) The inclusion of any ‘floating’ solutions not linked to a specific factor but considered by the group to be important; and (6) The group worked on a current and proposed daily schedule of times, activities and postures in a typical school day. Each focus group considered: (i) the current school day schedule, outlining ‘postures’ that students are currently involved in during the school day; and (ii) a proposed schedule, incorporating the groups’ ideas to “sit less and stand or move more”.

### Focus group methods

Focus groups were conducted by two trained research assistants (the research assistants had a minimum of PhD, Masters or Honours level Public Health or Dietetic Degree qualifications. They had no relationship to the participants), one facilitated the discussion and the other wrote notes capturing ideas and contextual information on a flipchart (large visible sheets of paper). The notes were in clear view of the focus group participants to allow them to review and reflect on their comments as they worked through each step of the ‘problem solution tree’. Participants contributed their ideas until no new ideas were forthcoming for each stage of the ‘problem/solution’ ideas generation. At the end of each stage participants were asked to review the notes on the large sheets of paper to ensure they reflected their ideas. All focus groups were also audio-recorded to allow researchers to seek clarity related to the notes if required.

### Thematic analysis

The themes were developed using participant ideas recorded on the flipchart during the focus groups. Initially two researchers familiarised themselves with the data by reading through the findings and making notes. Initial codes were identified then the researchers independently grouped the codes into potential themes. The researchers met to discuss and reach agreement on potential themes. A third researcher contributed to the discussion when finalising the themes. Appropriate titles for the final themes were developed to reflect the uniqueness of each theme.

## Results

**Fig. 1:**
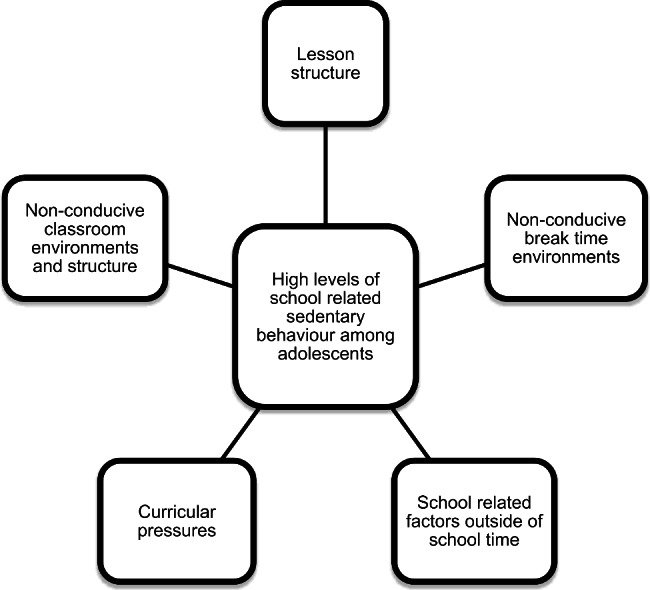
Thematic categories of identified ‘problems’

Informed consent was obtained from parents/guardians for 55 children (24 from Years 7 and 8. 13 males, 9 females and 2 gender unknown, and 31 from Years 9 and 10. 21 males, 10 females), and 31 teachers and school executives (14 males, 15 females and 2 gender unknown) (See Supplementary material 1, supplementary table 1). Thematic analysis resulted in five main categories of ‘problems’ across all groupings of the participants. The categories included: 1. Lesson structure; 2. Non-conducive classroom environments and structure; 3. Non-conducive break time environments; 4. Curricular pressures; and 5. School-related factors outside of the school environment (Fig. 1 and Supplementary material 3, supplementary table 2 and Supplementary material 4, supplementary table 3).

In general, students felt that most activities during the school day involved being sedentary, including those outside of class time (e.g. roll call/home room, assemblies and bus lines). Notably, some teachers and executives did not believe that student SB was a problem either in class or any other time during a regular school day. Teachers found it difficult to comprehend that there is a high rate of adolescent SB. For example, one teacher disagreed with the evidence stating that they constantly see active adolescents.

### Problem: lesson structure

When referring to the identified ‘problem’ (as per the problem/solution model) [22] of ‘lesson structure’, younger adolescents felt that lessons were designed to be sedentary where classwork is completed at their desk. This resulted in long periods of sitting (e.g. 6 periods of 50 min per regular school day). Participants believed being active and moving during class time was not a priority for teachers one student stated:

*“lessons are focused on academic effort, not activity”* (younger adolescent).

Periods of SB were exacerbated when there were behavioural issues during lessons resulting in additional periods of SB as teachers often prevented the whole class from commencing their break time as an alternative to punishing individual offenders. This not only increased periods of SB during the school day, but also resulted in fewer opportunities to be active during break times. In instances where students were punished individually this time was also usually spent being sedentary.

Older adolescents believed that double lessons (e.g. 1 h and 40 min) resulted in less movement between classrooms, increasing periods of SB. The students stated that ‘teaching style’ and ‘classroom control’ restricted classroom movement. Some relevant quotes include:

*“teaching style is textbooks and powerpoint,” “lessons are not formatted for standing”*, *“teachers like you to sit in classtime”.*

However, some students indicated they preferred to sit and write, or believed it was more efficient to sit when writing.

Teachers indicated that students were encouraged to sit as a means of controlling behaviour stating:

*“a good classroom is a quiet classroom” “easier to control students”,“sit means listen”*,

*“students are not settled when standing, it takes longer to learn”.*

Teachers also believed a crowded curriculum prevented activity during class time.

### Solutions: lesson structure

Significantly, proposed solutions to the ‘lesson structure’ problem from all three groups of participants included more ‘project-based’ activities as a means of reducing SB and promoting classroom movement. Student examples included:

*“active learning classes – standing and move around in maths” (younger adolescent)*

*“Move around more in classes, be more interactive – ‘act out’ English plays”(older adolescent)*

Younger adolescents proposed that punishment should include ‘moving’ with students using the following examples:

“picking up rubbish” or “being told to run around the school or do pushups”

They felt only offending individuals should be punished, rather than penalising other students, meaning the rest of the class could go outside to play. Teachers believed students and teachers needed a greater awareness and reminders about SB so that they would provide more opportunities for students to stand/move during lessons, including planned lessons which incorporate movement.

### Problem: Non-conducive Classroom Environment and structure

All groupings of participants provided similar examples for the second identified problem ‘classroom environment and structure’. Rooms were seen to be too small and desks difficult to move. The classroom layout with desks in rows was seen as the norm and was generally believed to promote SB. Participants remarked that furniture was not designed for standing and high student numbers meant classrooms were crowded allowing little scope for movement.

### Solutions: Non-conducive Classroom Environment and structure

When considering possible solutions all participant groups suggested extending classrooms and providing standing desks. Although, one teacher acknowledged that not all teachers supported the idea of standing desks as they believed them to be impractical. Teachers provided the following additional solutions: upright chairs or fitballs, ‘stretch’ or mid-lesson breaks, ‘changing the way lessons are taught’ and providing outdoor teaching spaces. Students suggested having sporting posters on walls, using portable technology (tablets, laptops), and increasing the number of active periods.

### Problem: non-conducive break time environments

All focus groups discussed the effect of social, policy or physical environments on break time SB during the school day (e.g., issues such as small break time areas, overcrowding on sports fields and the majority of surfaces being concrete).

Older students indicated they were restricted to ‘covered areas’ during break times preventing access to grass and playing fields. Clashes between year groups and a lack of shade on sporting fields were also identified as barriers to being active. Uniforms were seen to limit activity, older students describing uniforms as ‘uncomfortable’ and jackets as ‘heavy’, the requirement to wear school shoes (rather than sports shoes), one student believed ‘skirts were limiting’, and some students viewed their sports uniform as restrictive. Teachers and older students raised the hat policy as an issue (‘no hat, no play’) as it restricted opportunities to be active on playing fields. Older adolescents and teachers believed some students used environments such as the library as a safe place to retreat from other students (e.g. those who wanted to avoid being bullied), however, these environments promoted being sedentary during break times. Both teachers and older students believed students’ opportunities to be active were largely affected by pre-designated zones for school year groups. Teachers believed lunch breaks were too short to allow activity and school bags prohibited activity as they obstructed open areas and sporting fields.

Both groups of adolescents indicated students are conditioned to sit during break times citing plentiful seats, a lack of time to be active, an opportunity to talk with friends and the weather as reasons. Gender differences were noted by an older adolescent:

*“boys play handball and in the field at lunchtime; girls sit and talk”.*

Some students felt the social environment hindered break time activity stating:

*“if you don’t sit everyone looks at you – you are weird”(younger adolescent)*

These students stated that they often want to be active during break times but feel pressure not to. Others felt excluded from activities that occur during break times.

Technology (e.g. use of ipads) was considered a major barrier to PA during break times. The teachers and executive staff described the problem of technology as severe:

*“recent increase in ownership of technological devices has resulted in adolescents becoming more sedentary as they fear breaking their device if they move around”*.

The attraction of social media also increased the use of technology during break times.

Students identified physical environmental barriers that affected students’ opportunities to be more active. All groups believed a lack of facilities impeded opportunities for students to be active, describing a mix of problems. Playground spaces had a lack of equipment, too many people, and were largely concreted areas with limited grass. Students were not afforded access to the school hall where they could play sport (e.g. basketball). There were no changing facilities and showers were not permitted.

Younger adolescents believed classroom environments were a deterrent to activity when they came in from break times. Air conditioners were broken, blinds didn’t work and windows didn’t open. They felt ‘smelly and sweaty’ after sport. Additional barriers included a lack of variety in activity options during break times (e.g. handball or soccer), games being interrupted, the distance to playing fields and people sitting on playing fields which obstructed game play.

### Solutions: non-conducive break time environments

Teachers suggested students.

*“walk/talk rather than hangout at lunch”*.

Younger adolescents solutions included policies that ensured students go outside at lunch and are provided with opportunities to work in the garden. Additional suggestions included improving outdoor facilities (fake grass, plants), the provison of more playground space, addressing hygiene issues (open windows, deodorant, air fresheners, showers with privacy stalls), providing fixed playgrounds and sporting facilities, organising opportunities for more students to access areas ‘monopolised’ by other children, staggering oval access times, and compulsory activity (as *‘some are shy’*). Older adolescents suggested more flexibility with uniforms (e.g.,‘change into joggers at lunch’), being able to wear a Physical Education (PE) uniform to school and revising the materials used in uniforms. Teachers also recommended students bring a change of clothes or shoes for break times. Additional recommendations made by older adolescents included providing lockers, communal hats and showers, teachers assisting in organising games areas, and varying the games played to suit more students. The older adolescents and teachers mentioned changing the ‘no hat no play’ policy to a ‘no hat play in the shade’ policy.

### Problem: curricula pressures

The pressure to meet curriculum requirements may affect teachers’ inclination to provide regular breaks as stated by a younger student:

*“teachers feel they get less time if they give students breaks’*.

Older students believed this was indicative of the ‘pressure to perform academically’. Older students also noted they were required to sit for extended time periods doing exams (e.g. up to four hours of sitting).

Teachers and executive staff felt the extensive educational syllabus restricted time to meet curricula requirements, which could only be addressed when adolescent students are seated. They stated that subjects prioritised by education departments (Mathematics and English) are more conducive to being sedentary and were conducted more frequently, therefore promoting more SB. Further, teachers believed the:

*“board of studies did not like activity in learning unless there were evidence of outcomes”.*

Teaching pedagogies were described as:

“*more auditory and visual rather than kinetically focused”*.

### Solutions: curricula pressures

Teachers suggested there needed to be a change in the culture at the Department level in relation to exams.

### Problem: School-related factors outside of school time

Younger adolescents indicated that the time required to complete homework reduced the amount of time available to be active and limited opportunities for extra-curricular activities thus increasing SB. In addition, the environmental shift from smaller local primary schools to large secondary schools (located further from home), results in more motor vehicle travel time, again increasing time spent sedentary. Teachers reiterated this theme, stating that distance from school limited students’ opportunities to ride or walk to school. Teachers also felt their efforts to encourage students to reduce their SB was often hampered by a lack of follow-through in the home environment.

### Solutions: School-related factors outside of school time

Only younger students included a solution for this theme stating that a reduction in the amount of homework and assigning active homework could reduce SB outside of school hours.

### Imagine what a school day with 50% less SB would look like

When asked to imagine what a day with ‘50% less SB’ would look like, participants identified four main themes: (1) Make changes to the classroom and outdoor environments; (2) Make changes to the lesson structure and content; (3) Make changes to school policies; and (4) Introduce sporting equipment.

When discussing the Theme, *Changes to the classroom environment*, younger adolescents suggested classrooms with fewer chairs and tables and more benches; and older adolescents suggested rearranging the layout of desks/classrooms. One student indicated that it was not feasible to have 50% less SB, stating that it would be too noisy and that activity is an individual’s responsibility. Teachers’ suggestions included: work stations, activity pods, different furniture, larger classrooms and outdoor spaces, more useful outdoor and floor spaces, splitting classes between venues and smaller class sizes.

For the Theme, *Changes to the lesson structure*, younger adolescents suggested opportunities to go outside to learn, more ‘*hands-on activities’* and integrated non-active and active periods throughout the day. Older adolescents added: more opportunities to move in the classroom as part of learning, regular breaks during class time, standing at benches, and increased break time. Teachers suggested more upright hands-on short experiments with children rotating around the classroom, a blending of active learning in the board of studies curriculum, standing music lessons when using instruments, and active learning opportunities.

Ideas for the Theme, *School policy changes*, suggested by adolescents included active punishment – such as picking up papers (rather than being asked to stay sitting inside the classroom), having more comfortable uniforms or the option to wear a sports uniform to school, and double lessons of sport. Teachers suggested making PE classes compulsory.

For the Theme, *Introduce sporting equipment*, both younger and older adolescents suggested the need for more sporting equipment and teachers suggested more sports facilities and sports choices to cater for all interests, including having a pool and gym. Other suggestions included closing the library at lunchtime to prevent students from staying indoors and starting school earlier to allow more time across the day.

Additional ideas outside of these categories included the suggestion that teachers model standing and moving more. Teachers suggested starting the day with PA (e.g. morning walk) and running between classes. From a social perspective, it was suggested that students learn confidence-building exercises to change the current dynamics within the school environment which encouraged SB during break times. Students believed a school day with less SB would make them feel happier and healthier. Finally, a notable statement from the adolescent group was making ‘*being less sedentary feel normal’*.

## Discussion

This is the first known study that has used qualitative consultation with secondary school students and teachers to elicit feasible, practical, and translational ideas to encourage adolescents to “sit less and stand or move more” during the school day. Barriers to reducing school-based SB during adolescence (e.g., larger student numbers, fewer facilities and space), are likely different to those experienced during primary school [24]. Teachers’ and students’ contributions suggested policy, environmental, pedagogical and changes to social norms as feasible opportunities for adolescents to reduce SB throughout the school day.

The findings initiated similarities and differences in responses both between and across the groups of participants. Some teachers found it difficult to comprehend the high rates of adolescent sedentary behaviour, indicating a need for greater awareness, particularly in this cohort. All groups agreed classroom lessons were primarily sedentary, citing classroom control as a major contributor and offering project-based activities as a solution. The classroom environment was deemed too small, crowded and not conducive for movement suggesting physical changes to the classroom environment and pedagogical shifts as potential avenues for change. The breaktime environment, lacked facilities and older adolescents and teachers cited pre-designated playground zones, hat and library policies as problematic, while younger adolescents mentioned hygiene issues. Both adolescent groups identified gender differences and playground clashes as barriers and teachers talked about the impact of technology. Many of these problems could be addressed through policy interventions and changes to school routines, which are feasible low cost means of promoting behaviour change at the population level. Older adolescents and teachers reflected curricular pressures (at the Departmental level) and the pressure to perform promoting SB, while younger students referred to homework pressures. To prompt changes at the departmental level interventions need to demonstrate the benefit of reducing SB on learning outcomes.

Suggestions provided by teachers and students to reduce SB included changes to classroom layout/furniture, pedagogical changes, hands on learning, outdoor lessons, more comfortable uniforms, more breaks during class time, more compulsory PA, and access to outdoor equipment. These suggestions mirror recent initiatives in school redesign and a pedagogical shift encouraging more interactive classrooms, demonstrating favourable learning and wellbeing outcomes [14]. Recent primary school uniform research resulted in significant reductions in SB, illustrating the potential impact of policy changes, and showing promise for similar initiatives in the secondary school setting [25]. The fact that students and teachers in the current study considered the suggested initiatives feasible increases the likelihood of adoption into school settings, and subsequent improvements in adolescent SB. Co-designed interventions provide the opportunity for meaningful end user engagement providing context to intervention design [26]. Future research should therefore involve students and teachers in the co-design of interventions, partnering with them to consider the initiatives identified in this study as a starting point for intervention design [27].

Participants in the current study identified multiple suggestions to reduce SB in the school setting, including changes to classroom layout and furniture. To date most interventions designed to reduce SB in the secondary school setting have focused on ‘sit to stand’ desks as a solution to reduce classroom SB [28]. Few studies have targeted secondary school settings and pilot studies have had limited success in impacting health outcomes in adolescents [28]. While ‘sit to stand’ and ‘active’ (cycle) desks show promise for reducing classroom SB, previous research suggests that while adolescents’ perceptions of standing desks were favourable, they did not actively use the desks without being prompted by teachers [28]. This highlights the importance of recent recommendations promoting a whole-of-school approach to SB interventions and promoting adherence to school sitting recommendations [29]. The feasibility of their use with adolescents as a stand-alone solution to reduce SB could be questioned, particularly if it results in increased teacher burden [17].  Therefore it is crucial to consider both systematic and systemic approaches when planning and intervening in adolescent SB in the school context.

The diversity of ideas identified by the participants in the current study shows a need for a multidimensional approach to reduce adolescent SB. A recent shift in contemporary pedagogical approaches has resulted in the transformation of some school classroom spaces globally to accommodate the needs of students in the 21st century [12]. These new classroom designs have shifted from traditional forward-facing rows of desks to classrooms with areas of open space and a variety of furniture (e.g., soft chairs and cushions, writable walls, standing benches, open spaces, smaller nooks and the inclusion of technology) facilitating both individual and collaborative work that accommodates a range of postures (e.g. standing, lying, sedentary) and movement. The structure and pedagogies associated with these flexible classroom designs mirror the ideas suggested by students and teachers in the current study, where students and teachers suggested: active learning classrooms which incorporated standing and moving during class time, rearranging the layout of desks, more space in the classroom, interactive activities, the use of tablets to enable movement, allowing students to stand as an option, removing chairs, having more practical lessons, and a shift in mindset from students having to ‘sit’. More recent research indicates this type of classroom design improved adolescent sedentary profiles; promoting less overall SB, fewer prolonged bouts of SB, and more breaks in SB compared to adolescents in a traditional classroom (with rows of desks) [12]. This classroom design benefitted student interaction, engagement and collaboration, which may be important from a feasibility and translational perspective as community-based research requires collaboration and shared goals, acknowledging that the aims of the researcher may not be the priority of teaching staff.

Students in the current study felt they were conditioned to sit during the school day; particularly during break times. The students identified the need to oppose current social norms and normalise reducing SB during the school day, with one student indicating there was a need *to ‘make standing the norm*’. They believed teachers’ role modelling would assist in this transition. The lack of facilities, behavioural issues and social and gender expectations influenced break time movement behaviour choices (e.g.,girls sat and boys played handball). In many countries school break time can contribute approximately one hour of the day where adolescents could reduce SB and be more active [30]. Findings from this research indicate that girls in particular reported being conditioned to sit, which warrants further research. Students are frequently told to sit down as a means of controlling behaviour in the school setting, however there is a level of paradox as a growing body of evidence suggests PA has a positive effect on prosocial behaviour [31]. The social context and peer norms relating to SB in the school setting are largely under-researched. The findings from the current study suggest interventions aiming to reduce adolescent school-based SB and increase PA may be fruitless if social and peer factors are not considered and included as part of a multidimensional approach. Adopting a systematic and systemic lens when undertaking interventions to reduce adolescent SB in the secondary school context could improve research outcomes.

This study used a novel method to understand this complex issue and elicit potential solutions. It is the first known study that used qualitative consultation to elicit ideas to reduce adolescent SB during the school day. The study was limited by the fact that it involved a convenience sample of four independent schools. It is also limited by the fact that the data was collected between 2012 and 2014. However, considering there is a paucity of research investigating adolescent SB in secondary schools it is highly likely this research will make a contribution to future intervention design.

Investigating the influence of institutional settings (such as schools) is deemed to be one of the most modifiable contexts, however secondary school is a complicated setting with a multitude of influences and adolescence is a transition phase of development encompassing physical, emotional and cognitive changes. Initially interventions should aim to increase awareness relating to adolescent school based SB and possible solutions. Interventions could include physical environmental changes to the classroom environment coupled with supportive pedagogies and policy and departmental changes (which may have broader reach). The social context of adolescents’ schooling experience should also be considered when designing interventions. Future research should consider a dual systematic and systemic approach to navigate the complexity of adolescent SB in the school environment.

## Conclusions

With growing international interest in the school setting as an opportunity to reduce adolescent SB [32] this paper provides timely feedback revealing the thoughts and ideas of students and teachers. Importantly many of the suggestions from the focus groups seemed feasible. The social context of the school setting is also a crucial element of future study design (e.g., social norms). It is possible that education, policy changes, pedagogical support and some structural/environmental changes could result in ongoing improvements in adolescent SB.

## Electronic supplementary material

Below is the link to the electronic supplementary material.

Supplementary Material 1: Supplementary table 1. School and participant details

Supplementary Material 2: Focus group script

Supplementary Material 3: Supplementary table 2. Problems and solutions associated with adolescent school based sedentary time

Supplementary Material 4: Supplementary table 3. Themes identified by each target group

Supplementary Material 5: Standards for Reporting Qualitative Research (SRQR)

## Data Availability

The datasets used and/or analysed during the current study are available from the corresponding author on reasonable request.

## Abbreviations

LPA: Light Physical Activity
MET: Metabolic Equivalent of Task
MVPA: Moderate to Vigorous Physical Activity
SB: Sedentary Behaviour
HDL-C: High-Density Lipoprotein Cholesterol
HOMA2-S: Homeostasis Model Assessment 2
SBP: Systolic Blood Pressure
