# Supplementary material for: Making ‘being less sedentary feel normal’ –investigating ways to reduce adolescent sedentary behaviour at school: a qualitative study
Source: Int J Behav Nutr Phys Act. 2023 Jul 11;20:85. doi: 10.1186/s12966-023-01444-y (PMC10334559; doi:10.1186/s12966-023-01444-y)
Supplement: Supplementary file 1 — Supplementary Material 1: Supplementary table 1. School and participant details [file 12966_2023_1444_MOESM1_ESM.docx]

**Supplementary Table 1. School and participant details.**

| **School** | **School type** | **Approximate student numbers** | **Female**  **Teachers** | **Male**  **Teachers** | **Gender not identified** | **Total Teachers** | **Year 7 & 8 Female students** | **Year 7 & 8 Male students** | **Gender not identified** | **Year 9 and 10 Female students** | **Year 9 and 10 Male students** | **Total students** |
| --- | --- | --- | --- | --- | --- | --- | --- | --- | --- | --- | --- | --- |
| School A | Kinder to year 12. | 600 (300 high school) | 2 | 6 |  | 8 | 4 | 2 | 1 | 4 | 3 | 14 |
| School B | Kinder to year 12. | 240 (50 high school) | 2 | 1 |  | 3 | 1 | 2 |  | 4 | 2 | 9 |
| School C | Kinder to year 12. | 220 (120 high school) | 5 | 3 | 1 | 9 | 4 | 0 | 1 | 4 | 4 | 13 |
| School D | Single sex year 7 to 12. | 880 high school students | 6 | 4 | 1 | 11 | 0 | 9 |  | 0 | 10 | 19 |
