# Supplementary material for: Making ‘being less sedentary feel normal’ –investigating ways to reduce adolescent sedentary behaviour at school: a qualitative study
Source: Int J Behav Nutr Phys Act. 2023 Jul 11;20:85. doi: 10.1186/s12966-023-01444-y (PMC10334559; doi:10.1186/s12966-023-01444-y)
Supplement: Supplementary file 2 — Supplementary Material 2: Focus group script [file 12966_2023_1444_MOESM2_ESM.docx]

**Focus Group Script**

The research study you are participating in is titled ‘Investigating ways to reduce adolescent sitting time at school’

Today we are going to be talking about being sedentary.

**What does the word sedentary mean?**

Sitting while reading a magazine is a good example of a sedentary behaviour, but sitting while using a rowing machine at the gym would not count as sedentary behaviour as this activity requires effort.

A teenager/adolescent may obtain at least 60 minutes per day of moderate physical activity playing netball or soccer but can still be considered sedentary if they spend the majority of the rest of their day sitting or lying down, for example playing computer games.

Did you know that high school students spend more than 50% of their waking hours in sedentary behaviour?

And older adolescents (16-19 years) are the second most sedentary group in the population?

Who do you think is the most sedentary?

**Why might this be a problem?**

We know that teenagers/adolescents who are sedentary are more likely to be sedentary as adults. So we are hoping to make some changes to your level of activity now so that you may be more likely to be more active as adults.

Being sedentary means you might be less likely to keep fit and healthy as you get older. You might be more likely to have problems with your heart, be more likely to develop diabetes and some types of cancer or have a problem with your bones called osteoporosis.

So we would like to think we can make some changes to your school day that will help you sit less and stand more.

That’s why we are here today as a first step to solving this problem. We want to hear from you about what your school day looks like now in terms of how much time you spend sitting down each day.

Then we are going to ask you if you can think of some small changes in your school day that might make you spend less time sitting and more time being active.

Finally we will ask you what you think your school day might look like if your time at school was 50% more active than it is now.

We will be writing down what you say and also recording what you tell us. But we won’t write down your names. Anything you say during our discussion group will be anonymous (we won’t tell anyone the names of people who were part of the group.)
