# Supplementary material for: Making ‘being less sedentary feel normal’ –investigating ways to reduce adolescent sedentary behaviour at school: a qualitative study
Source: Int J Behav Nutr Phys Act. 2023 Jul 11;20:85. doi: 10.1186/s12966-023-01444-y (PMC10334559; doi:10.1186/s12966-023-01444-y)
Supplement: Supplementary file 3 — Supplementary Material 3: Supplementary table 2. Problems and solutions associated with adolescent school based sedentary time [file 12966_2023_1444_MOESM3_ESM.docx]

**Supplementary Table 2. Problems and solutions associated with adolescent school based sedentary time**

| **Themes (Problems)** | **Sub-problems** | **Solutions** |
| --- | --- | --- |
| Lesson structure | Long periods of sitting  Sitting is used to control the class and movement is not a priority  Curriculum design encourages sitting  Crowded curriculum and pressure to perform  Punishing the whole class and not just offenders | Modification of school day structure.  Greater awareness and reminders about SB.  Project based activities.  Active movement during class.  Timetabling active lessons between sedentary lessons.  Change of culture at the Departmental level  Only punishing offenders.  Active punishment (e.g., picking up rubbish, run around school). |
| Non-conducive classroom environment/structure | Small classrooms with fixed furniture in rows  Classroom layout /heavy furniture that promotes sitting | Larger classrooms.  Standing desks, upright chairs/fitballs.  Stretch breaks.  Portable technology.  Increased active periods.  Changing the way lessons are taught |
| Break time environment promotes SB | Restricted to school zones (by year group), over crowding  Lack of variety in options for activity during breaks  Clashes with fellow students  Lack of facilities, poor upkeep of facilities, too much concrete and no access to halls (with sporting facilities)  Uncomfortable uniform and lack of hats (restricting activity), personal hygiene following activity during breaks  People and school bags in the way on playing fields/open areas  Students accessing indoor environments (library)  Technology during break times  Conditioned to ‘sit and talk’  Gender differences girls sit and talk, boys play handball  Lunch breaks too short and distance to sporting fields was too far and people sat on the fields  Personal hygiene was a deterrent to activity and classrooms had broken air conditioners and windows that didn’t open | Organised opportunities to access areas and share access.  Staggered times to use sporting fields.  Teachers assisting to organise and provide variety in games  Fake grass, more playground space, fixed equipment, and sporting facilities.  Revise school uniforms.  Allow PE uniform to be worn to school.  Showers with privacy stalls.  No hat play in shade policy rather than no play.  Communal hats.  Access to lockers to allow a change of clothes and house school bags.  Close indoor environments that promote sitting  Ban technology during break times  Encourage ‘walk and talk’ |
| Factors affecting SB outside of school time | Amount of time to complete homework reduced extra-curricular activities  Moving from primary to high school resulted in more vehicle travel time  Lack of follow through in the home environment | Reduction in homework or active homework. |
| Curricular pressures | Pressure to meet curricular targets  Pressure for students to perform academically  Extended periods of SB as a result of long exams (up to 4 hours)  Extensive educational syllabus restricts time.  Educational departments prioritise more sedentary subjects (maths, English) | Change in the culture at the Departmental level in relation to exams. |

*Note: Solutions were not identified for all problems.
