# Supplementary material for: Making ‘being less sedentary feel normal’ –investigating ways to reduce adolescent sedentary behaviour at school: a qualitative study
Source: Int J Behav Nutr Phys Act. 2023 Jul 11;20:85. doi: 10.1186/s12966-023-01444-y (PMC10334559; doi:10.1186/s12966-023-01444-y)
Supplement: Supplementary file 4 — Supplementary Material 4: Supplementary table 3. Themes identified by each target group [file 12966_2023_1444_MOESM4_ESM.docx]

**Supplementary Table 3 Themes identified by each target group.**

|  | **Theme** | **Younger students** | **Older students** | **Teachers and executives** |
| --- | --- | --- | --- | --- |
| **Problem** | **Lesson structure** | Lessons designed to be sedentary | Double lessons resulted in less movement between classes increasing SB | Students are encouraged to sit as a means of control |
|  |  | Moving and being active is not a priority for teachers | Teaching style and classroom control restricted classroom movement | Crowded curriculum prevented activity during class time |
|  |  | Behavioural issues of one student prevented the whole class from starting break time |  |  |
|  |  | Individual punishment was spent being sedentary |  |  |
|  |  |  |  |  |
| **Solution** | **Lesson structure** | **Younger students** | **Older students** | **Teachers and executives** |
|  |  | Project-based activities | Project-based activities | Project-based activities |
|  |  | Punishment should include movement |  | Teachers and students need a greater awareness and reminders about SB |
|  |  | Only offending students should be punished |  | Planned lessons which incorporate  movement |
|  |  |  |  |  |
| **Problem** | **Non-conducive Classroom Environment and Structure** | **Younger students** | **Older students** | **Teachers and executives** |
|  |  | Rooms too small and desks difficult to move | Rooms too small and desks difficult to move | Rooms too small and desks difficult to move |
|  |  | Rows of desks promotes SB | Rows of desks promotes SB | Rows of desks promotes SB |
|  |  | Furniture is not designed for standing | Furniture is not designed for standing | Furniture is not designed for standing |
|  |  | High student numbers resulted in crowded classes with little room for movement | High student numbers resulted in crowded classes with little room for movement | High student numbers resulted in crowded classes with little room for movement |
|  |  |  |  |  |
| **Solution** | **Non-conducive Classroom Environment and Structure** | **Younger students** | **Older students** | **Teachers and executives** |
|  |  | Extending classroom size and providing standing desks | Extending classroom size and providing standing desks | Extending classroom size and providing standing desks |
|  |  | Sporting posters on walls |  | Not all teachers support the idea of standing desks as some see them as impractical |
|  |  |  |  | Upright chairs and fit balls |
|  |  |  |  | Stretch breaks or mid-less breaks |
|  |  |  |  | Change the way lessons are taught |
|  |  |  |  | Provide outdoor teaching spaces |
|  |  |  |  |  |
| **Problem** | **Non-conducive breaktime environment** | **Younger students** | **Older students** | **Teachers and executives** |
|  |  |  | Opportunities to be active affected by pre-designated zones for year groups | Opportunities to be active affected by pre-designated zones for year groups |
|  |  | Classrooms deterred activity at break time as they were in disrepair and were hot (broken air conditioning and inability to open windows) which promotes hygiene issues | Clashes between year groups and a lack of shade on sporting fields were barriers to being active |  |
|  |  | Felt smelly and sweaty after being active | Uniforms limit opportunities to be active | Technology is a severe problem which promotes SB |
|  |  |  | ‘No hat no play’ policy restricts opportunities to be active | ‘No hat no play’ policy restricts opportunities to be active |
|  |  |  | The use of the library as a ‘safe place’ to retreat from other students limits activity | The use of the library as a ‘safe place’ to retreat from other students limits activity |
|  |  | Students are conditioned to sit | Students are conditioned to sit |  |
|  |  | Perceived gender differences – boys are expected to be more active, girls are expected to sit | Perceived gender differences – boys are expected to be more active, girls are expected to sit |  |
|  |  | Lack of facilities and equipment, little grass and large concrete areas preventing activity | Lack of facilities and equipment, little grass and large concrete areas preventing activity | Lack of facilities and equipment, little grass and large concrete areas preventing activity |
|  |  | Lack of variety in activity options |  |  |
|  |  | Games being interrupted |  |  |
|  |  | Distance to playing fields |  |  |
|  |  | People sitting on playing fields and obstructing play |  | School bags obstruct activity on sporting fields |
|  |  |  |  |  |
| **Solution** | **Non-conducive breaktime environment** | **Younger students** | **Older students** | **Teachers and executives** |
|  |  |  |  | Students walk and talk rather than sit and talk during breaks |
|  |  | Improving outdoor facilities and providing fixed and non-fixed playground equipment | More flexibility with uniforms | Students bring a change of clothes for break time |
|  |  | More playground space | Provide lockers, communal hats |  |
|  |  | Addressing hygiene issues by opening windows, deodorant, air fresheners and shower stalls. | Provide showers |  |
|  |  | Organising opportunities for all students to access areas monopolised by other students | Teachers assist in organising games areas and varying games to suit more students |  |
|  |  |  | Change ‘no hat no play’ policy to ‘no hat play in the shade’ | Change ‘no hat no play’ policy to ‘no hat play in the shade’ |
|  |  |  |  |  |
| **Problem** | **Curricular pressures** | **Younger students** | **Older students** | **Teachers and executives** |
|  |  |  | Pressure to perform academically means teachers provide less breaks in sitting | Extensive syllabus could only be met if students were seated, influencing the amount of SB |
|  |  |  | Required to sit for long periods in exams | Subjects prioritised by education departments (Maths and English) are more conducive to SB and are conducted more frequently each week |
|  |  |  |  | The board of education did not like activity in learning unless there was evidence of outcomes |
|  |  |  |  | Pedagogies are auditory and visual rather than kinetic |
|  |  |  |  |  |
| **Solution** | **Curricular pressures** | **Younger students** | **Older students** | **Teachers and executives** |
|  |  |  |  | A change in culture at the Departmental level in relation to exams. |
|  |  |  |  |  |
| **Problem** | **School related factors outside of school time** | **Younger students** | **Older students** | **Teachers and executives** |
|  |  | Time required to complete homework limits extra-curricular activities and increases SB |  | Teachers’ efforts to discourage SB is hampered by a lack of follow through at home |
|  |  | Shift from smaller primary to large high schools increases travel time |  | Distance from school limits opportunities for students to walk or ride |
|  |  |  |  |  |
| **Solution** | **School related factors outside of school time** | **Younger students** | **Older students** | **Teachers and executives** |
|  |  | Reduce the amount of homework and include active homework |  |  |
